# Prevalence, patterns and associated risk factors for dyslipidaemia among individuals attending the diabetes clinic at a tertiary hospital in Central Malawi

**DOI:** 10.1186/s12872-023-03589-x

**Published:** 2023-11-09

**Authors:** Florence Filisa-Kaphamtengo, Jonathan Ngoma, Victoria Mukhula, Zaithwa Matemvu, Deborah Kapute, Peter Banda, Tamara Phiri, Mwapatsa Mipando, Mina C. Hosseinipour, Kondwani G. H. Katundu

**Affiliations:** 1 Kamuzu Central Hospital, Lilongwe, Malawi; 2 School of Global and Public Health, Kamuzu University of Health Sciences, Blantyre, Malawi; 3 Malawi-Liverpool Wellcome Clinical Research Program, Blantyre, Malawi; 4 Blantyre to Blantyre Research Facility, Kamuzu University of Health Sciences, Blantyre, Malawi; 5 Blantyre Adventist Hospital, Blantyre, Malawi; 6 Department of Medicine, Kamuzu University of Health Sciences, Blantyre, Malawi; 7 Department of Medicine, Queen Elizabeth Central Hospital, Blantyre, Malawi; 8 Department of Biomedical Sciences, Kamuzu University of Health Sciences, Blantyre, Malawi; 9 University of North Carolina Project-Lilongwe, Lilongwe, Malawi

**Keywords:** Dyslipidaemia, Atherosclerotic cardiovascular disease, Diabetes mellitus, Kamuzu central hospital, Malawi

## Abstract

**Background:**

Dyslipidaemia among individuals with diabetes is a significant modifiable risk factor for atherosclerotic cardiovascular diseases (ASCVDs). ASCVDs are a major cause of mortality and morbidity globally, especially in people with diabetes. In Malawi, limited data exist on the prevalence and biochemical characteristics of diabetic dyslipidaemia. This study investigated the prevalence and biochemical characteristics of dyslipidaemia in individuals attending the diabetes clinic at Kamuzu Central Hospital, the largest tertiary referral hospital in Central Malawi.

**Methods:**

Using a cross-sectional design, sociodemographic, medical and anthropometric data were collected from 391 adult participants who were enrolled in the study. Blood samples were analysed for glycosylated haemoglobin (HBA1c) and fasting lipid profiles. The prevalence of dyslipidaemia was calculated, and the biochemical characteristics of the dyslipidaemia were defined. The associations between dyslipidaemia and risk factors such as sociodemographic characteristics, obesity, and HBA1c levels were evaluated using logistic regression analysis.

**Results:**

Prevalence of dyslipidaemia was observed in 71% of the participants, and elevated low-density lipoprotein cholesterol was the most frequent lipid abnormality among the study participants. None of the participants were receiving any lipid-lowering therapy. On bivariate analysis, dyslipidemia was positively associated with female sex [OR 1.65 (95% CI 1.05- 2.58); *p* = 0.09], age ≥ 30 years [OR 3.60 (95% CI 1.17-7.68); *p* = 0.001] and overweight and obesity [OR 2.11 (95% CI 1.33-3.34); *p* = 0.002]. On multivariate analysis, being overweight or obese was an independent predictor of dyslipidaemia [AOR 1.8;(95% CI 1.15- 3.37); *p* = 0.04].

**Conclusion:**

Dyslipidaemia was highly prevalent among individuals with diabetes in this study, and elevated low-density lipoprotein cholesterol was the most frequent lipid abnormality. Overweight and obesity were also highly prevalent and positively predicted dyslipidaemia. This study highlights the importance of appropriately addressing dyslipidaemia, overweight and obesity among individuals with diabetes in Malawi and other similar settings in Africa as one of the significant ways of reducing the risk of ASCVDs among this population.

## Background

Approximately 537 million adults are living with diabetes mellitus (DM) globally, 75% of which live in low and middle-income countries (LMICs) [1]. In Malawi, the prevalence of DM is estimated at 7% [2]. DM is a significant risk for atherosclerotic cardiovascular diseases (ASCVDs), such as stroke, which is the major cause of morbidity and mortality in people living with DM [3]. ASCVDs cause 31% of all global deaths, with 80% occurring in LMIC [4]. Dyslipidaemia complicates DM and doubles the risk of cardiovascular events in individuals with DM [3, 5]. Dyslipidaemia is a driver of atherosclerosis and is an indirect cause of at least 2 million annual deaths and nearly 30 million disabilities globally [4].

Dyslipidaemia denotes a group of lipid abnormalities characterised by one or more of the following: elevated total cholesterol (TC), elevated low-density lipoprotein cholesterol (LDL-C), decreased high-density lipoprotein cholesterol (HDL-C), and elevated triglycerides (TG) [4, 6]. Diabetic dyslipidaemia is typically characterised by elevated triglycerides (TG), decreased high-density lipoprotein cholesterol (HDL-C) with normal to mildly elevated low-density lipoprotein cholesterol (LDL-C), owing to the overproduction of TG-rich very-low-density lipoprotein (VLDL) particles in the liver and increased exchange of TG in VLDL for cholesteryl esters in HDL and LDL-producing sdLDL [7–9].

There is limited data on the prevalence, biochemical characteristics, and risk factors of dyslipidaemia among individuals with DM in Malawi. This cross-sectional study aimed to determine the prevalence of dyslipidaemia, biochemical characteristics, and the associated risk factors among individuals with DM attending the adult DM clinic at Kamuzu Central Hospital (KCH), the largest tertiary referral hospital in Central Malawi.

## Methods

### Study design and setting

This quantitative cross-sectional study was conducted at the adult KCH, the largest tertiary referral hospital in Lilongwe, in central Malawi. KCH serves a population of about 7 million people in Central Malawi. The study population was adults aged 18 years and above with DM, either type 1 (T1DM) or type 2 (T2DM), irrespective of HIV status and attending the KCH DM clinic. Data were collected between March and June 2021.

#### Inclusion and exclusion criteria

Participants were included in the study if they were confirmed DM patients aged 18 years and older and consented to participate. We excluded pregnant participants, those with an incomplete medical history, those with fever or history of an active infection, and those from whom blood sample collection was unsuccessful.

#### Study population and sampling strategy

Study participants were enrolled when they visited the DM clinic. The study period coincided with the Coronavirus disease 2019 (COVID-19) pandemic and there were limited booked patients for the clinics. In that case, consecutive sampling was used to recruit participants in the study until the sample size was reached.

The clinical research nurse collected and documented data using an interviewer-administered questionnaire. Sociodemographic data were collected and recorded. Weight and height measurements were performed and recorded, from which the body mass index (BMI) was calculated. Clinical nurses also collected two blood samples in ethylenediaminetetraacetic acid (EDTA) tube for HBA1c tests and another in a plain tube for fasting serum lipid profile testing for LDL-C, TG, TC and HDL using an automated Erba XL640 (USA) by a qualified laboratory technologist.

#### Definition of parameters

Dyslipidaemia was defined as the presence of one or more lipid abnormalities among the study participants: TC > 200 mg/dl, LDL-C > 100 mg/dl, TG > 150 mg/dl and HDL-C < 40 mg/dl [10, 11]. Dyslipidaemia was further classified as isolated when a single abnormal lipid parameter (TC, TG, HDL-C or LDL-C) was present; combined when two lipid parameters (elevated TG, low HDL-C or elevated LDL-C) were detected; and mixed when all three lipid parameters are abnormal (elevated TG, low HDL-C and elevated LDL-C) [3]. Poor glycaemic control was defined as HBA1c of > 7% [10]. BMI categories were classified according to the WHO classification as underweight if BMI < 18.5, normal if BMI was between 18.5 and 25, overweight if BMI was between ≥25.0 and 30, and obese if BMI ≥ 30.0 [12].

#### Statistical analysis

Data were entered Microsoft Excel spreadsheet and statistical analysis was performed using STATA 17 (StataCorp LLC). The Shapiro- Wilks normality test was used to test the data for normality. Descriptive statistics for continuous data were expressed as the means or medians, and proportions for categorical data. The Chi-squared or Fisher’s exact test for independent variables was used to compare categorical data. The t-test was used to analyse the differences in mean difference in lipid concentrations between any two groups. Bivariate logistic regression was used to evaluate risk factors for dyslipidaemia, such as age groups, DM type, sex, or BMI categories. Multivariate logistic regression analysis accounted for confounding and included all variables with a *p* < 0.1 on bivariate analysis. In all cases, a *p-*value < 0.05 was considered significant.

## Results

### Participants recruitment process

**Fig. 1:**
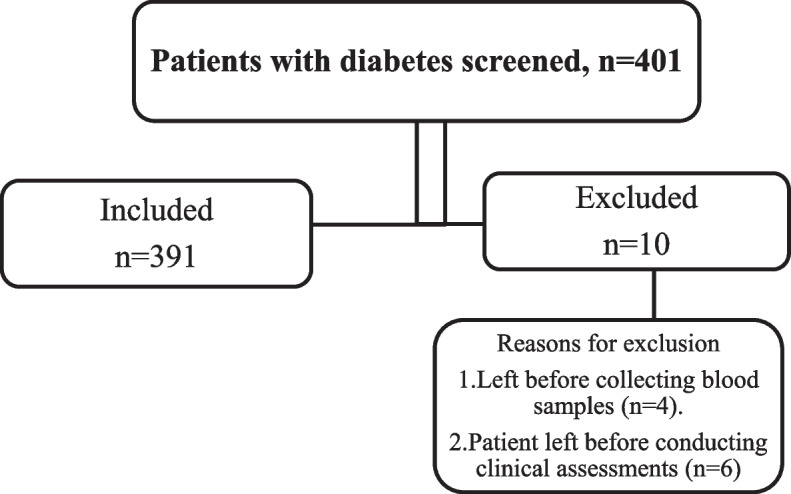
Strobe diagram illustrating the participant recruitment process, number of participants screened, inclusion and the reasons for exclusion in the study

A total of 401 participants were screened for inclusion in the study, and 391 were enrolled (Fig. 1). Participants were excluded from the study because they left before conducting clinical assessments or blood sample collection was not performed before blood pressure measurement (Fig. 1).

### Participant’s sociodemographic and clinical characteristics

Most of the study participants were females (64%), middle-aged with a mean age of 52 (± 13) years (Table 1). The HIV prevalence in the population was 11%, but the status was unknown in 13% of the participants. The mean blood pressure readings were 142 (± 26) mmHg systolic and 87.50 (± 15) mmHg diastolic. The mean HbA1c level was 10.29 (± 3.35).

**Table 1:** Participants’ characteristics

Variable	n (%)
Age category	
18-30	30 (8)
31-40	43 (11)
41-50	93 (24)
More than 50	225 (58)
Gender	
Male	141 (36)
Female	250 (64)
Education level	
None	23 (6)
Primary	163 (42)
Secondary	161 (41)
Tertiary	44 (11)
Occupation	
No formal employment	143 (36)
Farmer	57 (15)
Business	118 (30)
Formal employment	73 (19)
Type of DM	
Type 1	73 (19)
Type 2	318 (81)
Smoking Status	
Yes	4 (1)
No	387 (99)
Alcohol Consumption	
Yes	18 (5)
No	373 (95)
Intentional exercise intensity	
None	178 (46)
Minimum 30 minutes/day	138 (35)
More than 30 minutes/day	75 (19)
HIV Status	
Reactive	42 (11)
Non-Reactive	296 (76)
Unknown	53 (13)
Duration since diagnosis	
< 5 years	189 (49)
5-10 years	125 (32)
10.1-15 years	36 (9)
> 15 years	41(10)
High Blood Pressure Reading (≥ 140 mmHg systolic, ≥90 mmHg diastolic)	
No	174 (44)
Yes	222 (56)
HBA1c > 7%	
No	58 (15)
Yes	338 (85)

**Fig. 2:**
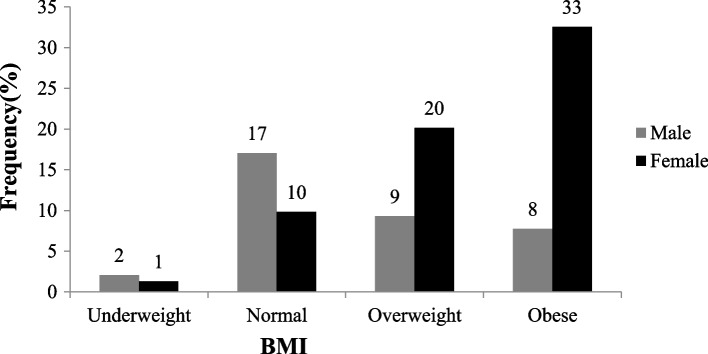
Body Mass index categories by sex. Female participants were more likely to be overweight and obese than the male participants [OR 5.32 (95% CI, 3.34 - 8.47); *p* < 0.001]

The prevalence of overweight and obesity in the study was 70% (30% overweight and 40% obese, respectively). Figure 2 shows the BMI categories classified by sex. Female participants were more likely to be overweight and obese than the male participants [OR 5.32 (95% CI, 3.34 - 8.47); *p* < 0.001].

### Prevalence of dyslipidaemia and the biochemical patterns in the study population

**Fig. 3:**
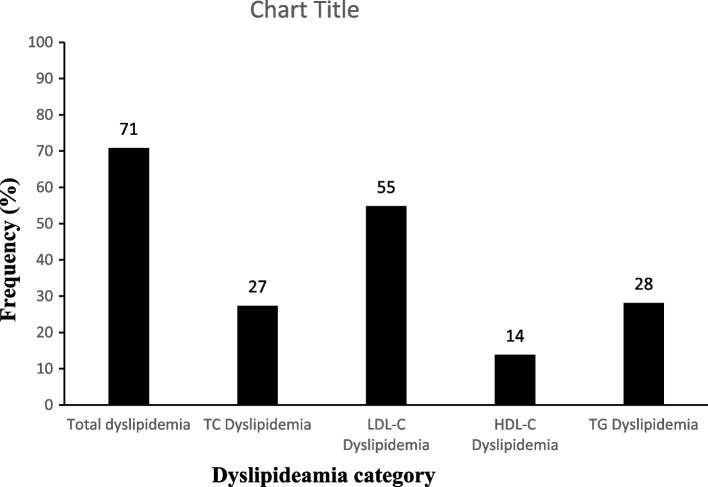
Frequency of lipid abnormalities in the study participants. Total dyslipidemia represents all individuals with dyslipidemia in study population. Elevated LDL-C dyslipidaemia was the most common lipid abnormality observed. The sum of the frequencies exceed 100% because most of the participants had a combination of at least two lipid abnormalities. TC = total cholesterol; LDL-C = low density lipoprotein cholesterol; HDL-C = high density lipoprotein cholesterol; TG = triglycerides

Dyslipidaemia was observed in 71% of the study participants (Fig. 3). Notably, elevated LDL-C concentrations were the most frequent lipid abnormality observed in 55% of the participants, and the least frequent type of lipid abnormality was decreased levels of HDL-C. At the time of the study, none of the individuals who participated were on lipid-lowering therapy.

Table 2 summarises the dyslipidaemia patterns regarding single, combined and mixed dyslipidaemia among participants who had dyslipidaemia, respectively. Isolated dyslipidaemia was the most common form, seconded by combined dyslipidaemia, and mixed dyslipidaemia was the least common pattern. In all the dyslipidaemia categories, elevated LDL-C was highly prevalent.

**Table 2:** Dyslipidaemia pattern distribution among study participants who had dyslipidaemia (*n* = 277)

Dyslipidaemia type	Parameter	N (%)
Isolated dyslipidaemia		**181 (65.34)**
	TC	7 (2.53)
	LDL	123 (44.40)
	TG	19 (6.86)
	HDL	32 (11.55)
Combined dyslipidaemia		**89 (32.13)**
	LDL + TG	70 (25.27)
	LDL + HDL	9 (3.25)
	HDL + TG	10 (3.61)
Mixed dyslipidaemia		**7 (2.53)**
	LDL + TG + HDL	7 (2.53)
Total		**277 (100)**

### Risk factors associated with dyslipidaemia among the study participants

Dyslipidaemia was positively associated with female sex, overweight and obesity (Table 3). T2DM was not statistically significantly associated with dyslipidaemia. Smoking status, alcohol consumption, education, occupation and HBA1c greater than 7% showed no significant association with dyslipidaemia in this study population. Overweight and obesity emerged as the independent predictor of dyslipidaemia [AOR 1.8 (95% CI 1.15- 3.37); *p* = 0.04] after adjusting for sex, age ≥ 30 years, physical exercise, and HIV status. Table 3 summarises these findings.

**Table 3:** Associated risk factors for dyslipidaemia in the study participants

Variable	Reference	Crude OR (95% CI)	*p*-value	Adjusted OR (95% CI)	*p*-value
Female sex	Male sex	1.65 (1.05 - 2.58)	0.029	0.74 (0.44 - 1.25)	0.25
Age ≥ 30 years	< 30 years	3.60 (1.17 - 7.68)	0.001	2.16 (0.92 - 5.08)	0.07
Diabetes type	Type 2	0.88 (0.39 - 1.14)	0.140	–	–
Alcohol consumption	No consumption	0.81 (0.30 - 2.20)	0.677	–	–
Physical exercise	Below WHO recommendation	0.50 (0.24 - 1.03)	0.061	0.46 (0.41-1.09)	0.06
HIV positive	HIV negative	1.84 (0.91 - 4.57)	0.07	2.11 (0.92-4.83)	0.08
Overweight & obese	Normal BMI	2.11 (1.33 - 3.34)	0.002	1.80 (1.02- 3.19)	0.04
HBA1c	Per 1% increase	1.00 (0.93 - 1.07)	0.972	–	–

## Discussion

This study at the largest tertiary referral hospital in central Malawi reports a high rate of dyslipidemia among adult individuals with diabetes, with elevated LDL-C as the most common lipid abnormality. Dyslipidaemia was positively associated with overweight and obesity, female sex, and age above 30 years. Overweight and obesity emerged as an independent predictor for dyslipidaemia among the study participants.

The study’s high prevalence of dyslipidaemia is comparable to other studies among individuals with DM in other Sub-Saharan African (SSA) countries [3, 10, 13]. These results are consistent with the previously reported high prevalence of dyslipidaemia at another public tertiary referral hospital in Malawi among individuals with DM and hypertension [14]. Dyslipidaemia is a significant modifiable determinant of ASCD globally and in Africa [5, 15] and requires proactive screening and management, especially among individuals at high risk, such as those living with DM. The absence of lipid-lowering therapy administration in this study highlights the lack of aggressive efforts towards addressing dyslipidaemia for individuals with DM in settings such as Malawi [14]. The substandard efforts toward addressing dyslipidaemia undoubtedly contribute to the escalating rates of ASCDs in the SSA [16].

Elevated LDL-C was the most common lipid abnormality in the present study. Elevated LDL-C-C drives atherosclerosis and is the target for treating dyslipidaemia [17, 18]. Therefore, the study participants were at higher risk for ASCDs, given the elevated levels of LDL-C, and should be treated with lipid-lowering therapy [18]. Adult individuals with DM between the ages of 40 and 75 years are recommended to be on statins for the primary prevention of ASCDs [18, 19]. More than 80% of the study participants were between 40 and 75 years old and ideally required at least moderate-intensity statin therapy [18, 19]. None of the participants in this study was on lipid-lowering therapy. Similar findings were reported at another public tertiary hospital in Malawi among individuals with DM and hypertension [14], indicating a lack of reinforced guidelines in addressing dyslipidaemia among this patient population in public health facilities of the country. Frequent stock-outs of lipogram reagents and unavailability of local guidelines for the management of dyslipidaemia are significant barriers to efforts towards addressing dyslipidaemia among people with DM in Malawi and other LMICs [14, 20, 21]. It is imperative, therefore, that the Ministry of Health in Malawi and its partners reinforce efforts toward strategies for optimally addressing dyslipidaemia among patients with DM to reduce the risk of ASCDs.

Overweight and obesity were highly prevalent (70%) in this study and independently predicted dyslipidaemia. The rates of overweight and obesity among the participants were higher than the 40% rate reported in the general population in urban Malawi [22]. The difference may be because the present study involved participants with DM, of whom 81% had T2DM, for which overweight and obesity are known risk factors [23]. However, given the study’s cross-sectional nature, it was complex to establish the temporal relationship between DM, overweight and obesity and dyslipidaemia. Similar to our results, other African studies have reported overweight and obesity as independent predictors of dyslipidaemia [4, 6, 13]. Socio-economic transitions such as urbanisation influence sedentary lifestyles and poor dietary habits, promoting overweight and obesity in Africa and Malawi [22, 24]. Recent data suggest that more than two-thirds of the Malawi adult population do not consume recommended adequate amounts daily of fruits and vegetables [25]. Adequate consumption of whole fruits and vegetables is essential for better BMI control and normal nutrition [26], and should be encouraged, especially among people with DM [26].

Females in this study were more likely to be overweight and obese than men, as reported in Malawi [22] and other African countries [23]. Overweight and obesity in women in Malawi, like in many African countries, are influenced by the socio-cultural context where it is revered and regarded as a sign of high economic standing, beauty and a sign of fertility [23, 27, 28]. Additionally, in urban settings, women are engaged mainly in jobs requiring less physical activity. In Lilongwe city, the risk of obesity is expectedly high [27, 29]. Overweight and obesity must be addressed among individuals with DM to curb dyslipidaemia and the risk of ASCDs in this population [9]. Malawi has in-country-trained dieticians working at tertiary-level healthcare facilities and should reinforce lifestyle modifications, including proactive physical activity and appropriate cardiovascular-friendly dietary habits, towards reducing the rates of overweight and obesity among individuals with DM [30, 31].

Poor glycaemic control, as indicated by HBA1c ≥7%, was observed in 85% of participants in the present study. These results are similar to previous studies in Southern Africa [10]. If left unmanaged, poor glycaemic control positively influences dyslipidaemia [32] and increases the risk of cardiovascular events for every 1% increase in HBA1c level [33]. These considerations necessitate re-strategising the monitoring and delivery of efficacious interventions like dietary adjustments, glucose monitoring and physical exercising to help improve glucose control.

The interplay of obesity among individuals with DM, dyslipidaemia and poor glycaemic control in this study is significant as it relates to increased cardiovascular risk. A complex interaction of biochemical parameters has been described relating how obesity, genetic predisposition, oxidative stress, and inflammatory markers relate to diabetic dyslipidaemia and hence mediate CVDs [34–36]. Diabetic dyslipidaemia positively influences oxidative stress, a pro-inflammatory environment, with higher inflammatory markers such as C-reactive protein and 4-hydroxynonenal adducts and cellular damage hence influencing ASCDs [35, 37]. Additionally, obesity and dyslipidaemia in DM have been associated with lower serum adiponectin levels, a decreased expression of genes expressing adiponectin such as rs2241766 and rs1501299 and their respective receptors [34, 36]. Furthermore, obesity in DM is linked to higher leptin levels which influences endothelial dysfunction and the development of ASCDs [38]. Strategies towards decreasing the prevalence of dyslipidaemia, obesity and poor glycaemic control are, therefore, imperative to reduce cardiovascular risk in this population.

Although smoking and alcohol consumption are known risk factors for dyslipidaemia, there was no statistically significant association with dyslipidaemia in this study. The prevalence of smoking and alcohol consumption in this study was lower than NCD STEPwise survey findings (1 and 5% versus 11.2 and 17%, respectively) [25]. The lack of significant association would have likely been due to the lack of statistical power, owing to the low prevalence of the conditions in this study. Moreover, the participants may have underreported since they are advised to abstain from alcohol and smoking at the DM clinic during health education sessions.

The HIV prevalence in the study population was 11%. A previous study in Malawi showed that the rates may be as high as 20% among people with DM [14]. Dyslipidaemia and DM increase the risk of ASCDs up to 2.4-fold in people living with HIV (PLWH) [19, 39–42]. In this study, the association of HIV and dyslipidaemia approached significance, and low power may have influenced the lack of statistical significance due to the low prevalence of HIV among the participants. Nevertheless, it is essential to carefully consider the choice of lipid-lowering therapy in PLWH with DM and dyslipidaemia [43]. Atorvastatin is the preferred choice of statin among PLWH, unlike simvastatin and rosuvastatin, due to the high risk of drug interactions with antiretroviral drugs [43, 44]. Clinicians must evaluate potential drug-drug interactions between lipid-lowering agents and ARVs before prescription [43, 45].

This study had its limitations. The cross-sectional nature may not establish a cause-and-effect relationship. Nonetheless, our study complements previous findings on the high prevalence of diabetic dyslipidaemia in Malawi at tertiary hospitals and informs the need for clinical guidelines on screening and management of diabetic dyslipidaemia at facilities in Malawi.

## Conclusion

This study highlights the high prevalence of diabetic dyslipidaemia characterised by a high frequency of elevated LDL-C, with overweight and obesity as an independent positive predictor. These results reinforce the need to proactively address dyslipidaemia, overweight and obesity among individuals with DM in LMICs as one of the significant ways of reducing the risk of ASCDs among this population group.

## Data Availability

The datasets used and/or analysed during the current study are available from the primary author upon reasonable request.

## Abbreviations

ASCVD: Atherosclerotic cardiovascular disease
BMIq: Body mass index
CI: Confidence interval
COMREC: College of Medicine Research and Ethics Committee
COVID-19: Coronavirus disease 2019
DM: Diabetes mellitus
EDTA: Ethylenediaminetetraacetic acid
GCP: Good clinical practice
HBA1c: Glycosylated haemoglobin
HDL-C: High-density lipoprotein cholesterol
HIV: Human immunodeficiency virus
KCH: Kamuzu central hospital
LDL-C: Low-density lipoprotein cholesterol
OR: Odds ratio
ARR: Adjusted odds ratio
T1DM: Type 1 diabetes mellitus
T2DM: Type 2 diabetes mellitus
TC: Total cholesterol
TG: Triglycerides
USA: United States of America
VLDL: Very low-density lipoprotein
WHO: World Health Organisation
